# 757. Bridging the Gap: An Educational Intervention to Increase HIV PrEP Access in Resident Primary Care Clinics

**DOI:** 10.1093/ofid/ofad500.818

**Published:** 2023-11-27

**Authors:** Jennifer M Davis, Daniel A Solomon, Erik K Alexander, Subha Ramani

**Affiliations:** University of Nebraska Medical Center, Omaha, Nebraska; Brigham and Women's Hospital / Harvard Medical School, Boston, MA; Brigham & Womens Hospital, Boston, Massachusetts; Brigham and Women's Hospital and Harvard Medical School, Boston, Massachusetts

## Abstract

**Background:**

HIV Pre-Exposure Prophylaxis (PrEP) is a safe and effective way to prevent HIV acquisition. Yet, in 2020, only 25% of individuals in the US and Puerto Rico who had an indication for PrEP received a PrEP prescription. While PrEP is accessible to primary care clinicians, many do not feel comfortable prescribing PrEP. Clinician education has been identified as a key opportunity to increase access and comfort in prescribing PrEP.

**Methods:**

An educational intervention was designed aiming to increase PrEP knowledge and prescribing among residents and preceptors in the primary care clinics at Brigham and Women’s Hospital (BWH). The intervention followed the six steps described in Kern’s model of curriculum development (Figure 1). After formal needs assessment, an in-person interactive teaching session was designed and presented. This was supplemented by an online reference guide and note template dot phrases accessible in the electronic medical record (EMR). The curriculum was evaluated with pre and post intervention surveys. Survey data were analyzed using descriptive statistics and one tailed T tests.

**Figure:**
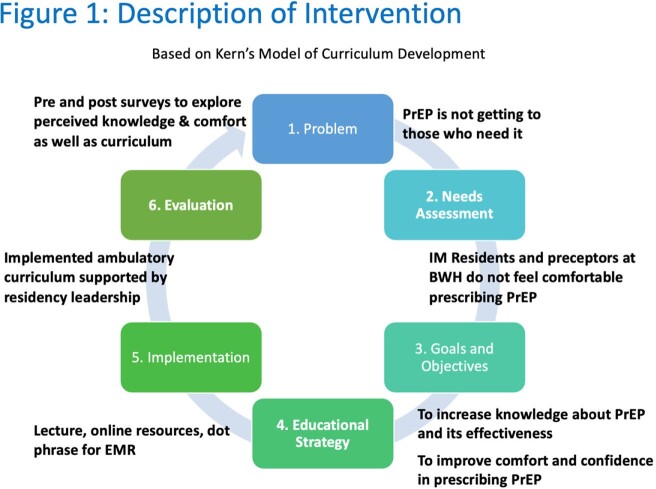

**Results:**

Of the approximately 30 residents and preceptors who participated in the intervention, 19 completed the pre survey and 9 completed the post survey. Only 37% (7/19) of pre survey respondents reported having had PrEP education in the past. 21% (4/19) had prior teaching during medical school, 21% (4/19) during residency, and 16% (3/19) in continuing medical education courses. Participants indicated that their comfort with prescribing PrEP increased overall after the intervention with a statistically significant increase in reported confidence in their ability to prescribe PrEP (mean Likert scale value of 3.88 vs 2.95, p = 0.03) (table 1).

**Figure:**
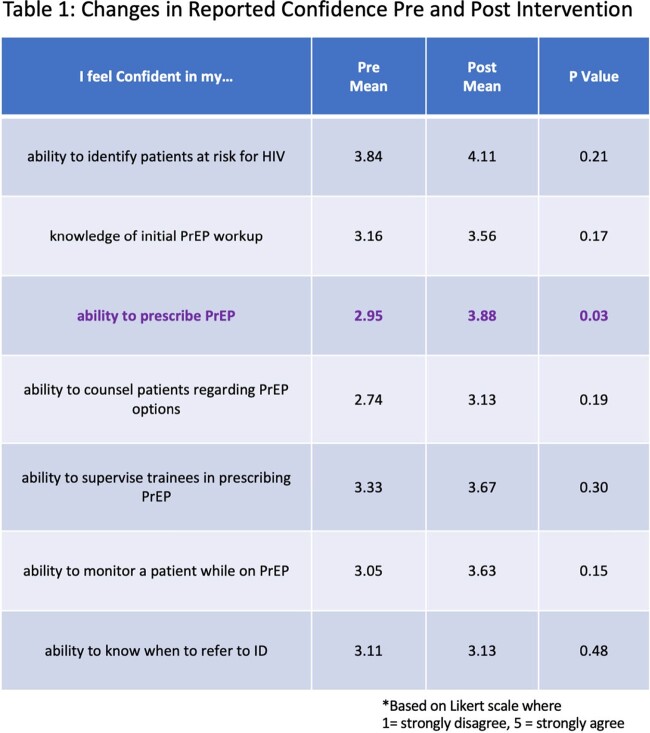

**Conclusion:**

PrEP is an underutilized modality in preventing HIV transmission, but many primary care clinicians do not feel comfortable prescribing PrEP even though it is accessible in primary care settings. Systematic PrEP education is lacking in the UME, GME, and CME settings. Targeted, interactive, multimodal educational interventions can be effective in increasing comfort with PrEP prescribing. Support from leadership and learner buy-in is critical for effective training.

**Disclosures:**

**All Authors**: No reported disclosures

